# Diagnostic Performance of Multiple Sound Touch Elastography for Differentiating Benign and Malignant Thyroid Nodules

**DOI:** 10.3389/fphar.2018.01359

**Published:** 2018-11-26

**Authors:** Lei Zhang, Zhimin Ding, Fajin Dong, Huaiyu Wu, Weiyu Liang, Hongtian Tian, Xiuqin Ye, Hui Luo, Jinfeng Xu

**Affiliations:** ^1^Department of Ultrasound, First Affiliated Hospital of Southern University of Science and Technology, Shenzhen Medical Ultrasound Engineering Center, Shenzhen People’s Hospital, Shenzhen, China; ^2^Department of Ultrasound, Second Clinical College of Jinan University, Shenzhen People’s Hospital, Shenzhen, China; ^3^The Key Laboratory of Cardiovascular Remodeling and Function Research, Chinese Ministry of Education, Chinese Ministry of Health, The State and Shandong Province Joint Key Laboratory of Translational Cardiovascular Medicine, Qilu Hospital of Shandong University, Jinan, China

**Keywords:** Sound Touch Elastography, thyroid cancer, shear modulus, summary receiver operating characteristic curve, diagnosis

## Abstract

This study evaluated the ability of Sound Touch Elastography (STE) to distinguish malignant from benign thyroid nodules by quantifying tumor stiffness using the elastic ratio (EI) and shear modulus (G). Eighty-six patients with 86 nodules were enrolled in this study. There were 24/86 (27.90%) thyroid papillary carcinomas (TPC) and 62/86 (72.10%) benign nodules. The mean EI was significantly lower in TPCs than in benign nodules. The EI area under the receiver operating characteristic curve (ROC) was 80%. The EI cutoff value for TPCs was 0.215%. The sensitivity (Sen), specificity (Spe), positive likelihood ratio (LR+), and negative likelihood ratio (LR-) were 71%, 73%, 2.58, and 0.40, respectively. *G*_max_, *G*_mean_, and *G*_sd_ were significantly higher in TPCs than in benign nodules. There were no significant differences in *G*_min_. Compared with other G parameters, *G*_max_ with an optimal cutoff value of 15.82 kPa had the highest AUROC value (84%). The Sen, Spe, LR+, and LR- were 79.17%, 79.03%, 3.776, and 0.261, respectively. We pooled the EI, *G*_max_, *G*_mean_, and *G*_sd_ and the pooled-Sen, Spe, LR+, LR-, diagnostic odds ratio and odds ratio, and area under the summary ROC were 79%, 71%, 2.73, 0.29, 2.23, 9.29, and 82%, respectively. STE could be a new ultrasound diagnostic method for evaluating benign and malignant thyroid nodules.

## Introduction

Thyroid cancer (TC) is the most well-known type of endocrine-related malignancy; TC has become threefold more common in the past 30 years (Cooper et al., 2006; Sebag et al., 2010; Saranac et al., 2011). Fine-needle aspiration (FNA) plays a critical role in differentiating thyroid nodules because of its high sensitivity and specificity (Paschke et al., 2011). However, it is invasive and involves high non-diagnostic (10–15%) or indeterminate (10–20%) outcomes (Rago et al., 2010).

Ultrasound elastography is a non-invasive tool, first introduced by Ophir et al. (1991), which has shown promise for the evaluation of tissue stiffness, providing additional and clinically relevant information. Mapping the stiffness can either be estimated by analyzing the strain in tissue under stress (quasi-static method) or by imaging of shear waves, mechanical waves, whose propagation is governed by tissue stiffness rather than by its bulk modulus (Gennisson et al., 2013). Most malignant nodules are portrayed by the organization of their unusually firm stroma because of the features of collagen and myofibroblasts, which enable the identification of thyroid cancers with elastography imaging (Monpeyssen et al., 2013).

Nowadays, Sound Touch Elastography (STE) has emerged as a novel elastography technique, which can both provide maps of the strain and of the shear waves. STE allows for quantification of tissue stiffness with the elastic ratio (EI) and shear modulus (G) using the same ultrasound equipment. The main aim of our study was to evaluate the usefulness of STE in predicting malignant thyroid nodules using histopathological analysis as the reference standard.

## Materials and Methods

### Study Population

This study was approved by the Ethics Committee of the Shenzhen people’s Hospital. Between June 2016 and December 2017, 95 patients referred for ultrasound examinations were recruited for this study. All participants signed the informed consent form required by the human study committee before enrollment. Nine participants were lost to follow up. The inclusion criteria were as follows. (1) The nodules were stable when detected by ultrasound (US), (2) the size of the nodules ranged from 0.5 to 3.0 cm, (3) the nodules appeared solid or almost solid (<20% cystic) on US, (4) sufficient thyroid tissue surrounded the nodules at the same depth and US cross-section, (5) no intervention or surgery on the nodules had been performed before the US examination, and (6) thyroid surgery or fine needle aspiration biopsy (FNAB) was performed after the US examination within 1 week. Not every nodule could be included for the patients with multiple nodules in this study because of the size and component of nodule restrictions. For patients who had multiple nodules, only the nodule that best satisfied the inclusion criteria was included. Finally, 86 patients (mean age 46.43 ± 12.17 years, range 26–78 years) with 86 nodules (mean size 1.60 ± 0. 39 cm, range 1.01–2.98 cm) met the inclusion criteria and were enrolled in this study, including 24 thyroid papillary carcinomas (TPC) and 62 benign nodules. Thirty-eight patients underwent FNAB and 28 underwent surgery in our hospital.

### Imaging Techniques

Both conventional US and STE examinations were performed with a Resona 7 ultrasound system (Mindray Medical Solutions, ıShenzhen, China) equipped with a 11L3 linear array transducer (bandwidth frequency of 3–11 MHz) and STE software. When the patients met the inclusion criteria, an informed consent was obtained. Patients were in a supine position with a fully exposed neck. Following which, an STE examination of the lesion was performed. First, the examination probe was kept in contact with the skin. Next, the ROI size was adjusted, such that both the lesion and enough surrounding tissues were included in the ROI and the majority of the lesion’s longitudinal section was included in the center of the ROI. When the left of the dual images was nearly green (area >95%) of ROI, the elastographic image of the lesion was acquired and saved. The nodule’s morphology characteristics, size, boundary, echoes, and color Doppler features were observed by conventional US and stored. First, switching to EI of the STE model, the probe touched the skin lightly, adjusting the size of the region of interest (ROI), ensured that the nodule and sufficient surrounding gland tissue were included in the ROI. The maximum longitudinal section of the nodules was displayed at the center of the screen, and then the patients were instructed to hold their breath, when the green control bar at the bottom of the screen was stable, pressed the update button and the image was saved; then, the same method was used to obtain the short axis section. Second, switching to the shear wave model, the patient maintained the same position without breathing, when the left of the dual images is nearly green (area >95%) of the ROI, the update button was pressed to obtain the long and short axis section images.

Elastography images were produced, the nodules were circled, and the EI and G data (including *G*_max_, *G*_min_, *G*_mean_, and *G*_sd_) were obtained. The average of the long-axis and short-axis values were used for further statistical analysis.

All the examinations were conducted by a sonographer with more than 7 years of experience in US and 3 years of experience in elastography and who was blind to the histopathological results.

### Pathological Diagnoses

All nodules were confirmed by histology in this study. All pathological diagnoses were performed by a pathologist with more than 8 years of experience in thyroid pathological examination.

### Statistical Analysis

The STE data were recorded, including EI, *G*_max_, *G*_min_, *G*_mean_, and *G*_sd_. The true-positive (TP), true-negative (TN), false-positive (FP), and false-negative (FN) numbers per method were calculated. The Kruskal–Wallis non-parametric test was used to compare whether there were significant differences in the EI, *G*_max_, *G*_min_, *G*_mean_, and *G*_sd_ between benign and malignant nodules. The abilities of EI, *G*_max_, *G*_min_, *G*_mean_, and *G*_sd_ to differentiate malignant from benign nodules were evaluated by receiver-operating characteristic (ROC) curve analysis. The best cutoff values were obtained using the Youden index (sensitivity+specificity-1) from the ROC curve analysis. The sensitivity (Sen), specificity (Spe), positive likelihood ratio (LR+), and negative likelihood ratio (LR-) were calculated using the chi-squared test (χ^2^-test). A *P*-value < 0.05 was considered to be statistically significant. The values were then combined to obtain an overall STE analysis. The area under the summary ROC (SROC) curve was used. The pooled Sen, Spe, LR+, and LR- were calculated. These data were analyzed with Stata 14.0 for Mac (Stata Corp, College Station, TX, United States) and GraphPad Prism 6.0 for Mac (GraphPad Software, Inc., San Diego, CA, United States).

## Results

### Histology

**FIGURE 1:**
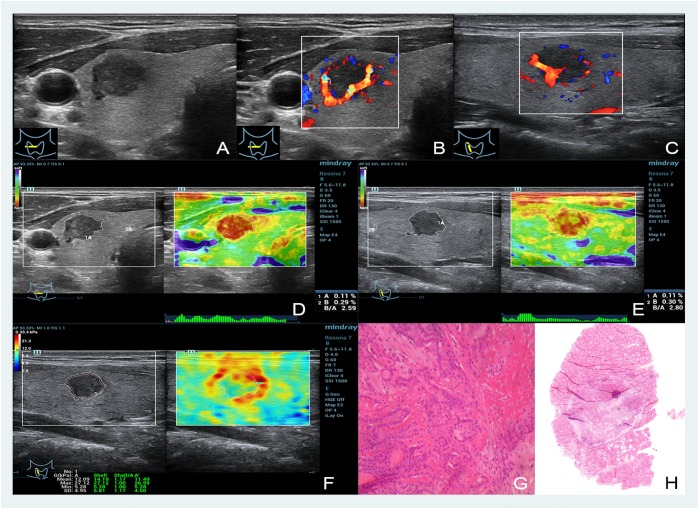
Images showing a thyroid papillary carcinoma in a 48-year-old man. **(A–C)** The B- and color-mode images show a hypoechoic nodule by ultrasound, visible blood flow signal around; **(D,E)** short and long axis of the nodule evaluated by EI (the strain ratio both are 0.11%); **(F)** quantitative shear wave values (*G*_mean_: 12.09 kPa, *G*_max_: 27.12 kPa, *G*_sd_: 4.95 kPa) were measured by drawing a ROI around the nodule to encompass the maximum area but not include the tissue outside the nodule displayed on the B-mode image; **(G,H)** the pathology results confirmed that the nodule was a thyroid papillary carcinoma.

**FIGURE 2:**
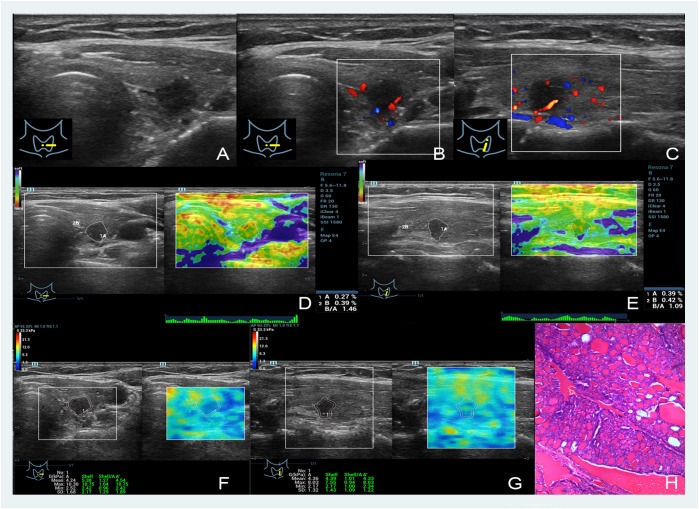
Images showing a nodular goiter in a 46-year-old woman. **(A–C)** The B- and color-mode images show a hypoechoic nodule by ultrasound, minor visible blood flow signal around and inside; **(D,E)** short and long axis of the nodule evaluated by the EI (the strain ratios are 0.27 and 0.39%, respectively); **(F,G)** quantitative shear wave values (*G*_mean_: 4.24 kPa, *G*_max_: 10.30 kPa, *G*_sd_: 1.68 kPa in the short axis; *G*_mean_: 4.39 kPa, *G*_max_: 8.03 kPa, *G*_sd_: 1.32 kPa in the long axis) were measured by drawing a ROI around the nodule to encompass the maximum area but not include the tissue outside the nodule displayed on the B-mode image; **(H)** the pathology results confirmed that the nodule was a nodular goiter.

The pathology results revealed that among the nodules (Figures 1A–C,G,H), there were 24/86 (27.90%) malignant papillary carcinomas and 62/86 (72.10%) benign nodules (46 goiter nodules and 16 adenomas) (Figures 2A–C,H).

### EI of Benign and Malignant Nodules

The nodules’ stiffness was indicated by different color changes. As shown in Figures 1D,E and Figures 2D,E, blue in the upper left indicates the softest and red indicates the hardest. In this study, 24 malignant nodules were red (20 nodules were bright red, which occupied the entire nodule, and the other 4 cases of red area more than 80%), and 62 benign nodules had similar color: green or yellow and white.

**FIGURE 3:**
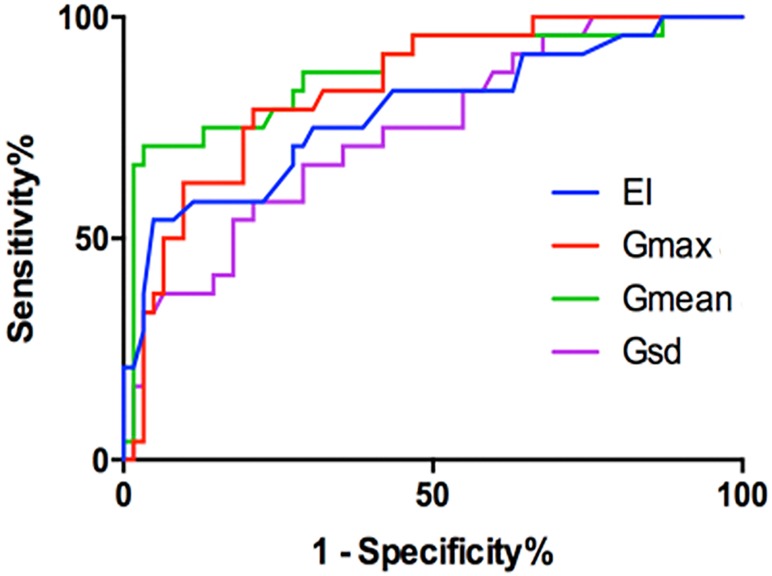
The ROC of the EI (cutoff: 0.215%, Sen 71%, Spe 73%), *G*_max_ (cutoff: 15.82 kPa, Sen 79%, Spe 79%), *G*_mean_ (cutoff: 6.715 kPa, Sen 86%, Spe 68%), and *G*_sd_ (cutoff: 2.00 kPa, Sen 78%, Spe 64%).

The EI measurements of benign and malignant nodules are shown in Table 1. The mean EI of malignant nodules was statistically lower than that of benign nodules (0.19 ± 0.02% vs. 0.29 ± 0.01%, *P* < 0.001). The area under the ROC curve (AUROC) for the EI of thyroid nodules for the diagnosis of malignant nodules was 0.8. The cutoff value of the EI for malignant nodules was 0.215%. Using this cut-off value, the TP, FP, FN, and TN were 17, 17, 7, and 45, respectively. The Sen, Spe, LR+, and LR- of the EI for diagnosing malignant nodules were 71%, 73%, 2.58, and 0.40, respectively (Figure 3 and Table 1).

**Table 1:** The EI and G measurements of benign and malignant nodules.

Lesion		MAX	MIN	Mean ± SD	*P*	Cut-off	AUROC	SEN	SPE	LR+	LR-	TP	FP	FN	TN
EI	Benign	0.78	0.13	0.29 ± 0.01	0.0001	0.215%	0.78	0.71	0.73	2.58	0.40	17	17	7	45
	Malignant	0.34	0.07	0.19 ± 0.02											
*G*_mean_	Benign	0.51	0.13	5.82 ± 0.30	0.0006	6.715	0.78	0.86	0.68	2.71	0.19	21	20	3	42
	Malignant	0.34	0.07	8.22 ± 0.78											
*G*_max_	Benign	49.36	4.48	12.12 ± 0.99	0.0001	15.82	0.84	0.79	0.79	3.78	0.26	19	13	5	49
	Malignant	41.97	8.03	21.62 ± 1.65											
*G*_min_	Benign	5.61	0.14	2.64 ± 0.17	0.5905	–	–	–	–	–	–	–	–	–	–
	Malignant	8.48	0.45	2.83 ± 0.37											
*G*_sd_	Benign	0.51	0.13	1.75 ± 0.15	0.0002	2.00	0.74	0.78	0.64	2.21	0.34	19	22	5	40
	Malignant	0.34	0.07	2.92 ± 0.30											


*SEN, sensitivity; SPE, specificity; LR+, positive likelihood ratio; LR-, negative likelihood ratio; TP, true-positive; TN, true-negative; FP, false-positive; FN, false-negative.*

### G of Benign and Malignant Nodules

The nodules’ stiffness was indicated by different color changes like EI. As shown in Figures 1F,G, 2F,G, blue in the upper left indicates the softest and red indicates the hardest. In this study, all 24 malignant nodules showed a reddish periphery (red rim-like) and a yellow or yellow-green in the middle, while the color of 62 benign nodules was similar to that of the surrounding tissues and was either green or yellow and had no red nodules with malignant nodules.

The G measurements of benign and malignant nodules are shown in Table 1. *G*_max_, *G*_mean_, and *G*_sd_ were significantly higher in malignant nodules than in benign nodules (*P* < 0.005) (Table 1). There were no significant differences in *G*_min_ (*P* = 0.59). The ROC curves of the three G parameters are shown in Figure 3. The optimal cut-off values with respective AUC are presented in Table 1. Compared with other G parameters, *G*_max_ with the optimal cutoff value set at 15.82 kPa had the highest AUC value, the AUROC was 0.840 (95% CI: 0.759–0.928). Using this cutoff point, showing diagnostic Sen, Spe, LR+, and LR- were 79.17%, 79.03%, 3.776, and 0.261, respectively (Table 1). The other two G parameters, *G*_mean_ and *G*_sd_, with the same cutoff value were 6.715 and 2.00 kPa, respectively. The details are shown in Table 1 and Figure 3.

### Pooled EI and G of Benign and Malignant Nodules

**FIGURE 4:**
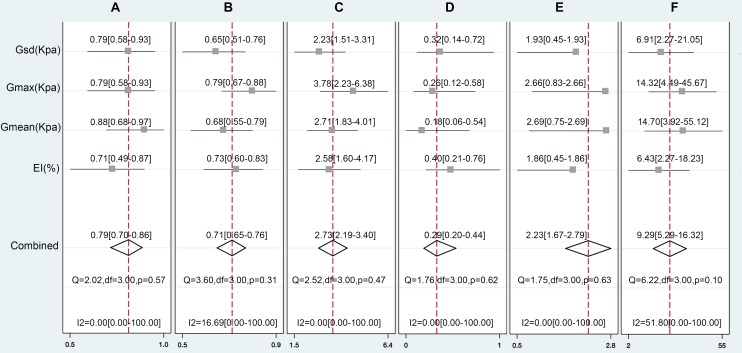
The pooled Sen **(A)**, Spe **(B)**, LR+**(C)**, LR-**(D)**, DOR **(E)**, and OR **(F)** of the EI, *G*_max_, *G*_mean_, and *G*_sd_ with 95% CI.

**FIGURE 5:**
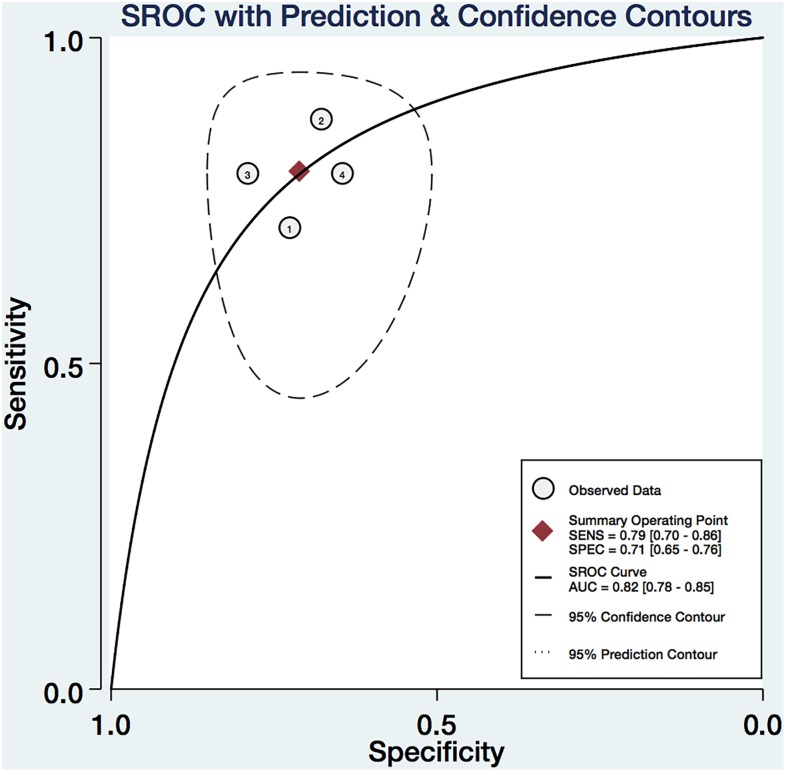
The SROC of the EI, *G*_max_, *G*_mean_, and *G*_sd_ with 95% CI.

We pooled the EI, *G*_max_, *G*_mean_, and *G*_sd_ using the Midas module in Stata 14.0, which is equipped with the bivariate mixed-effects regression model developed by van Houwelingen et al. (1993, 2002), modified for the synthesis of diagnostic test data, to pool the statistical indexes and draw the statistical graphs. The pooled sensitivity (P-Sen) and specificity (P-Spe), pooled positive likelihood ratio (PLR+), negative likelihood ratio (PLR-), diagnostic odds ratio (DOR), OR with corresponding 95% confidence intervals (CI), and area under the SROC curve were used to examine the diagnostic accuracy. As shown in Figure 4, significant heterogeneity in P-Sen (*I*^2^ = 0%, *Q* = 2.02) and P-Spe (*I*^2^ = 16.69%, *Q* = 3.60) were detected. The P-Sen, P-Spe, PLR+, PLR-, DOR, OR, and area under the SROC curve were 79% (95% CI: 70–86%), 71% (95% CI: 65–76%), 2.73 (95% CI: 2.19–3.40), 0.29 (95% CI: 0.20–0.44), 2.23 (95% CI: 1.67–2.79), 9.29 (95% CI: 5.29–16.32), and 82% (95% CI: 78–85%), respectively (Figures 4, 5).

## Discussion

Approximately 60,000 people a year will be diagnosed with thyroid cancer in the United States. Statistics show that more women than men are diagnosed with thyroid cancer. It is the fourth most common type of cancer among women (National Comprehensive Cancer Network [NCCN], 2017). Tumor stiffness can reflect the nature of tumors to a certain extent (Mandel, 2004). The harder the tissue, the higher the risk of malignancy (Pacini et al., 2006). Overall, cancerous thyroid tumors tend to be harder than benign ones. Nodule stiffness is normally determined by palpation by a physician, but smaller nodules, particularly those that are deeply located, cannot be palpated.

Elastography is a non-invasive ultrasound method for assessing the mechanical qualities of the tissues, for example, elasticity and stiffness (Bamber et al., 2013). Since the thyroid is a superficial organ, it can undoubtedly be assessed by all elastography techniques. Elastography has been generally acknowledged and utilized for distinguishing the stiffness of thyroid nodules (Franchi-Abella et al., 2013). Different techniques, based on real-time two-dimensional image sequencing after applying a force that is either dynamic or slowly varying (and considered “quasi-static”), were developed. The principle of elastography is based on US measurement of tissue displacement (Gennisson et al., 2013). All elastographic techniques utilize signal processing to create an image and/or to evaluate the stiffness and elasticity of the investigated tissue (Golu et al., 2017). Two basic concepts are currently used for US elastography: to evaluate the strain or distortion of a tissue because of a power (static elastography, SE), the propagation speed of a shear wave (acoustic radiation force impulse imaging, ARFI), or the measurement of the Young’s modulus of the tissue (shear wave elastography, SWE). Manufacturers have built their elastography modules utilizing any of these principles and their own particular innovations.

All shear wave data in the entire ROI could be received at one time to achieve real time two-dimensional SWE imaging. STE can obtain the quantitative elastic parameters, EI and G, and non-invasively evaluate tissue stiffness. In this study, the diagnostic and clinical values of the EI and G of benign and malignant thyroid nodules were compared. The cut-off points of the EI, *G*_max_, *G*_mean_, and *G*_sd_ were 0.215% (Sen: 71%, Spe: 73%), 15.82 kPa (Sen: 79%, Spe: 79%), 6.715 kPa (Sen: 86%, Spe: 68%), and 2.00 kPa (Sen: 78%, Spe: 64%), respectively. A meta-analysis reported the diagnostic value of elastography in predicting malignant thyroid nodules. Tian et al. (2016) reported results indicating that real-time elastography (RTE) was more accurate than SWE as suggested by higher Sen, Spe, and AUROC. The P-Sen, P-Spe, and SROC of RTE and SWE were: 82.9% (95% CI: 79.9–85.5%), 82.8% (95% CI: 78.9–86.2%), and 88.9% and 78.4% (95% CI: 73.2–82.8%), 82.4% (95% CI: 76.6–87.1%), and 85.9%, respectively. We also performed a meta-analysis on ARFI for differentiating thyroid nodules (Dong et al., 2015). A total of 13 cohort studies involving 1617 thyroid nodules from 1451 patients were identified. Of 13 studies, one was retrospective and the others were prospective. The pooled Sen, Spe, LR+, LR-, and DOR of SWE in differentiating malignant from benign thyroid nodules were 86.3% (95% CI: 78.2–91.7), 89.5% (95% CI: 83.3–93.6), 7.04 (95% CI: 4.40–11.26), 0.17 (95% CI: 0.10–0.31), and 46.66 (95% CI: 19.47–111.81), respectively. The area under the SROC curve was 94% (95% CI: 92–96). In this study, we also pooled the data by systematic review and obtained P-Sen, P-Spe, and area under the SROC curve at 79% (95% CI: 70–86%), 71% (95% CI: 65–76%), and 82% (95% CI: 78–85%), respectively.

**FIGURE 6:**
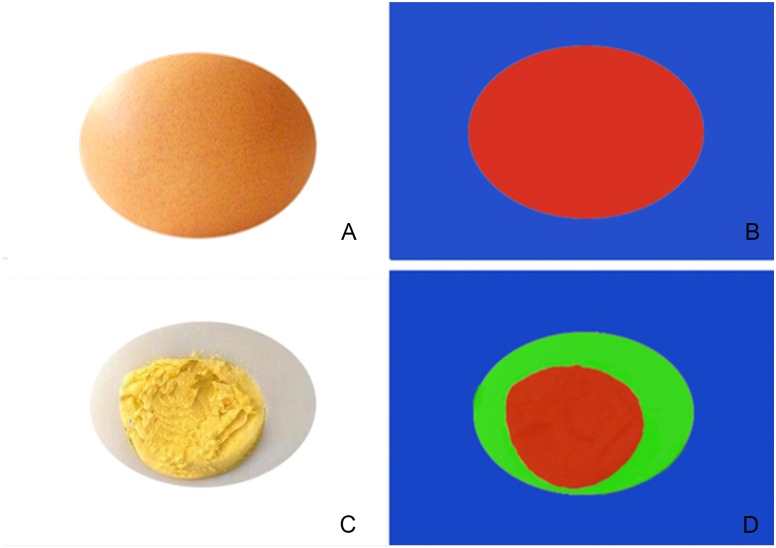
Simple schematic diagram of EI **(A,B)** and shear wave imaging **(C,D)**, **(A,B)** show the shelled raw eggs, when a force is applied, it will cause a slight deformation, but internal deformation would not be detected, and therefore, the machine would interpret that the nodules are all hard, in EI with pure red. **(C,D)** Show in shear wave imaging, the probe emits shear waves into the nodule that are received by the probe, and can detect in-depth the hardness of different components inside the nodule and display, similar to detecting a peeled cooked egg which could display various internal.

Regarding why on the STE elasticity image, the malignant nodules of EI showed a red nodule, the SWE images showed a red rim-like, and the inside were the same color as the benign nodules. We believe that this is attributable to the different principles of the two imaging methods. The EI is a stain-force imaging system. After an external force to the tissue which would cause deformation. Unlike other elastography modalities, STE does not need to be pressurized by the probe, which could monitor the third party’s pulsating blood vessels and breathing. Regarding the nodule stiffness, for example, of shelled raw eggs (Figures 6A,B), when a force is applied, it will cause a slight deformation, but internal deformation would not be detected, and therefore, the machine would interpret that the nodules are all hard, in EI with pure red. In shear wave imaging, the probe emits shear waves into the nodule that are received by the probe, and can detect in-depth the hardness of different components inside the nodule and display, similar to detecting a peeled cooked egg (Figures 6C,D), which could display various internal. The details of stiffness were shown in different colors. During the invasive growth of malignant tumors, internal necrosis is often accompanied by shear wave elasticity. This is termed the “stiff-rim” sign, consistent with the relevant reports (Zhou et al., 2014). An increase in the stiffness of the surrounding tissue may signify that cancer cells have invaded the surrounding tissue of the tumor (Itoh et al., 2006). Studies have shown that tumor tissue infiltration around the tumor is an independent prognostic factor of tumor recurrence and patient death (De Mascarel et al., 1998).

These results suggested that tissue stiffness detected by STE can excellently reflect the nature of the nodules. Moreover, STE has some advantages. First, the EI and G can be obtained with the same ultrasound equipment with the same probe; second, the elastography images can be stored in the equipment and analyzed later, which reduces the examination time and the patient’s discomfort. However, the study still had limitations. First, the number of cases was small, further study is needed with a larger sample size. Second, only the EI and G were evaluated by ROC curve analysis in this study, and in the further research, E and V and edge infiltration should be also evaluated.

## Conclusion

The present study showed comparable results for the novel quantitative STE for the differentiation of thyroid nodules. With high Sen and Spe, STE could be recognized as a new ultrasound diagnostic method in evaluating benign and malignant thyroid nodules. However, the sample of this preliminary study may not be representative of a screening population. Multicenter prospective studies with non-surgical populations and larger samples and more noteworthy difference of pathology are warranted.

## Author Contributions

FD guarantees the integrity of the entire study and contributed to the experimental studies. JX and ZD contributed to the study concepts. FD and ZD designed the study. LZ contributed to the literature research. FD and HW contributed to the clinical studies. HW acquired the data. FD and LZ contributed to the data analysis and interpreted the data. FD and HT contributed to the statistical analysis. XY prepared the manuscript. WL and ZD contributed to the intellectual content of the manuscript. FD and XY edited, revised, and reviewed the manuscript.

## Conflict of Interest Statement

The authors declare that the research was conducted in the absence of any commercial or financial relationships that could be construed as a potential conflict of interest.
